# Morphologies and prognostic significance of left ventricular volume/time curves with cardiac magnetic resonance in patients with non-ischaemic heart failure and left bundle branch block

**DOI:** 10.1007/s10554-021-02194-3

**Published:** 2021-02-26

**Authors:** Alberto Aimo, Alessandro Valleggi, Andrea Barison, Sara Salerni, Michele Emdin, Giovanni Donato Aquaro

**Affiliations:** 1 Scuola Superiore Sant’Anna, Piazza Martiri della Libertà 33, 56124 Pisa, Italy; 2 Fondazione Toscana Gabriele Monasterio, Piazza Martiri della Libertà 33, 56124 Pisa, Italy; 3 University Hospital of Chieti, Chieti, Italy

**Keywords:** Cardiac magnetic resonance, Left bundle branch block, Dyssynchrony, Prognosis

## Abstract

Patients with non-ischaemic systolic heart failure (HF) and left bundle branch block (LBBB) can display a wide or narrow pattern (WP/NP) of the systolic phase of the left ventricular (LV) volume/time (V/t) curve in cardiac magnetic resonance (CMR). The clinical and prognostic significance of these patterns is unknown. Consecutive patients with non-ischaemic HF, LV ejection fraction < 50% and LBBB underwent 1.5 T CMR. Maximal dyssynchrony time (time between the earliest and latest end-systolic peaks), systolic dyssynchrony index (standard deviation of times to peak volume change), and contractility index (maximum rate of change of pressure-normalized stress) were calculated. The endpoint was a composite of cardiovascular death, HF hospitalization, and appropriate defibrillator shock. NP was found in 29 and WP in 72 patients. WP patients had higher volumes and NT-proBNP, and lower LVEF. WP patients had a longer maximal dyssynchrony time (absolute duration: 192 ± 80 vs. 143 ± 65 ms, p < 0.001; % of RR interval: 25 ± 11% vs. 8 ± 4%, p < 0.001), a higher systolic dyssynchrony index (13 ± 4 vs. 7 ± 3%, p < 0.001), and a lower contractility index (2.6 ± 1.2 vs 3.2 ± 1.7, p < 0.05). WP patients had a shorter survival free from the composite endpoint regardless of age, NT-proBNP or LVEF. Nonetheless, WP patients responded more often to cardiac resynchronization therapy (CRT) than those with NP (24/28 [86%] vs. 1/11 [9%] responders, respectively; p < 0.001). In patients with non-ischaemic systolic HF and LBBB, the WP of V/t curves identifies a subgroup of patients with greater LV dyssynchrony and worse outcome, but better response to CRT.

## Background

Cardiac magnetic resonance (CMR) provides morphologic and functional information relevant to a broad array of cardiovascular disorders. Its main qualities are excellent spatial and temporal resolution, unrestricted tomographic fields, and no exposure to ionizing radiation. CMR is the gold-standard technique for the quantification of left ventricular (LV) volumes, and offers a variety of alternative applications for the assessment of both systolic and diastolic function, some of them superior to echocardiography in accuracy and reproducibility, other complementary [1, 2].

Several techniques for the evaluation of LV dyssynchrony by CMR have been proposed [3]. Conventional analysis of the short-axis, balanced steady-state free precession (bSSFP) acquisition allows to calculate LV volume/time curves and their first derivative dV/dt across all cardiac phases; cardiac dyssynchrony can be assessed by visualizing how steep the ventricular emptying is and how it is distributed throughout systole. Sohal et al. introduced the systolic dyssynchrony index, defined as the standard deviation of the regional times to peak volume change from segmental volume/time (V/t) curves [4]. Further analyses may be performed by tracking myocardial deformation during the cardiac cycle and calculating systolic strain and strain rate. Feature tracking analysis of bSSFP imaging detects anatomical features of interest in the LV subendocardium and subepicardium along the cardiac cycle, similarly to echocardiographic speckle tracking [5, 6]. A second technique is cardiac tagging, which is based on the application of a specific radiofrequency pulse at baseline to mark several lines or grids in the myocardium, which can then be followed over time; it and has been validated against sonomicrometry measurements [7]. A circumferential uniformity ratio estimate (CURE) derived from myocardial tagging has been proposed [8]. A third technique relies on phase contrast imaging, which can be used to track myocardial movements in any direction with almost the same frame rates as echocardiography. This technique remains to be properly validated, as a close relationship with echocardiographic and hemodynamic data was demonstrated only in small studies [9, 10]. Other techniques to calculate strain and strain rate have been investigated [11, 12].

**Fig. 1:**
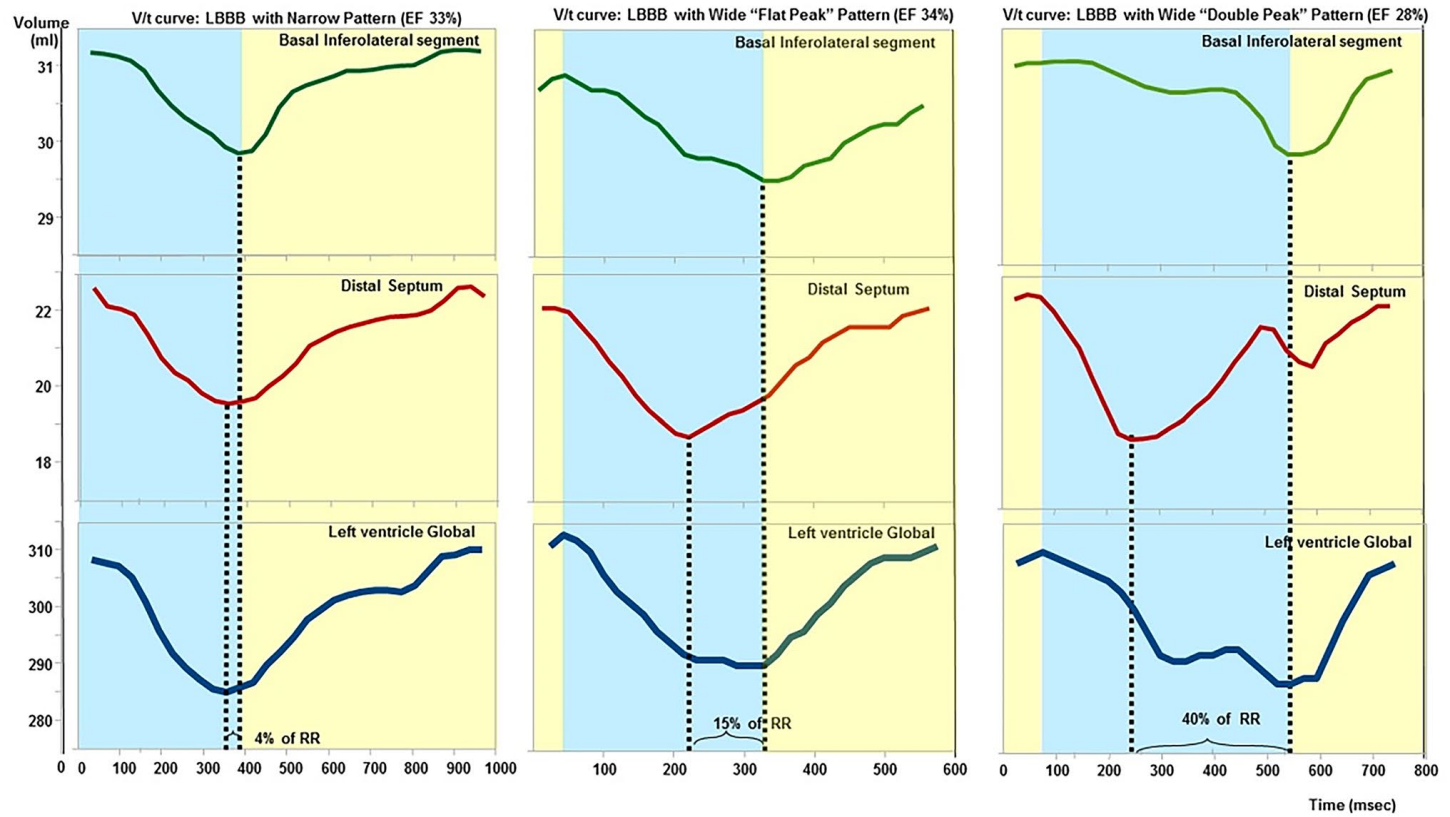
Examples of narrow pattern and wide pattern. The volume/time (V/t) curves of 3 patients with non-ischaemic systolic heart failure are reported. The regional V/t curve of basal inferolateral region and distal septum are shown in the upper and middle panel, respectively. The panel below provides the global left ventricular V/t curves. The maximal dyssynchrony time was 4% of the R-R interval in the patient with the narrow pattern (*left*), 15% in the patient with the wide, “flat-peak” pattern (*centre*), and 40% in the patient with the wide, “double-peak” pattern (*right*)

Among these possible approaches to the assessment of LV dyssynchrony, V/t curves are particularly promising because they can be automatically generated from standard bSSFP images by common post-processing CMR software. V/t curves visually represent the changes in LV volumes throughout the cardiac cycle, and could be potentially used to perform a quantitative assessment of both systolic and diastolic function [13]. We observed that, in patients with non-ischemic HF and left bundle branch block (LBBB), the systolic phase of the V/t curve can display two patterns: a narrow pattern (NP), similar to the profile of subjects with no LBBB, and a wide pattern (WP), showing an irregular profile and multiple peaks (Fig. 1). This study aimed to define the clinical correlates of the WP pattern, its impact on patient outcome, and on the response to CRT.

## Methods

### Study population

Consecutive patients with non-ischaemic systolic HF (LVEF < 50%) and LBBB evaluated in the outpatient clinic of a tertiary referral centre for HF (Fondazione Toscana Gabriele Monasterio, Pisa, Italy) were referred to CMR examination at the same Institution. CMR was requested as part of a comprehensive screening of chronic HF patients (see below). The inclusion criteria were: a diagnosis of HF according to European Society of Cardiology Guidelines [14, 15]; clinical stability and no changes in therapy since ≥ 3 months; non-ischaemic aetiology; LBBB; no contraindications to CMR examination. All patients underwent coronary angiography to establish the non-ischaemic aetiology, in agreement with the World Health Organization definition (“a dilated cardiomyopathy with impaired contractile performance not explained by the extent of coronary artery disease or ischemic damage”) [16]. Exclusion criteria were: age < 18 years, congenital heart disease, recent myocarditis (< 6 months), peripartum cardiomyopathy, arrhythmogenic right ventricular cardiomyopathy, severe primary valve disease, untreated hypertension, hypertrophic cardiomyopathy, cardiac amyloidosis, estimated glomerular filtration rate < 30 mL/min/1.73 m^2^, and contraindications to CMR (severe claustrophobia, presence of CMR-unsafe metal devices). Furthermore, patients with “benign” aetiologies such as enolic, Tako-tsubo or tachymocardiopathies were excluded. LBBB was diagnosed through standard, 12-lead electrocardiogram (ECG), following established criteria: QRS duration ≥ 130 ms; QS or rS in lead V1; broad (frequently notched or slurred) R waves in leads I, aVL, V5, or V6; and absent q waves in leads V5 and V6 [17].

101 patients were prospectively enrolled from 2013 to 2015. In addition to 12-lead ECG and CMR (see below), these patients underwent:

- Transthoracic echocardiogram, performed and interpreted according to current Guidelines [18–22];
- Laboratory evaluation: full blood cell count, C-reactive protein, creatinine, transaminases, thyroid hormones, cardiac troponin I, N-terminal pro-B-type natriuretic peptide (NT-proBNP), resting norepinephrine (NE), plasma renin activity, and aldosterone levels) [23];
- Symptom-limited cardiopulmonary exercise testing (CPET) on a bicycle ergometer (Vmax, Sensormedics, Yorba Linda, CA, USA) [24].
- 24-hour ECG Holter recording by a three-lead (precordial, posterior, inferior leads) digital system (Elamedical, France). At Holter monitoring, ventricular ectopic beats (VEBs) were considered relevant if they were grade IV or V on the Lown scale [25].

Informed consent was obtained from each patient. The study protocol conformed to the ethical guidelines of the 1975 Declaration of Helsinki [26], and was approved by the Institution’s human research committee. Patients were compared with 20 age- and sex-matched healthy controls, undergoing CMR with no contrast medium administration.

### Cardiac magnetic resonance

Patients underwent CMR with an 8-channel phased-array surface receiver coil and vectorcardiogram triggering using a 1.5 T scanner (Signa Excite, GE Healthcare, Milwaukee, USA). Biventricular systolic function was assessed by breath-hold steady-state free precession cine imaging in the short-axis (SA) stack (8-mm thickness, no gap). Sequence parameters were: field-of-view: 360–400 mm, repetition/echo time: 3.2/1.6 ms, flip angle: 45–60°, matrix: 224 × 224, phases: 30. Late gadolinium enhancement (LGE) imaging was performed between 10 and 20 min after contrast agent administration (Gadoteric acid, DOTAREM, 0.2 mmol/kg) using a segmented T1-weighted gradient-echo inversion-recovery pulse sequence. In SA orientation, the LV was encompassed by contiguous 8-mm thick slices (with no inter-slice gap). Inversion time (TI) was individually adapted to suppress the signal of normal remote myocardium (220–320 ms). Sequence parameters were: field-of-view: 360–400 mm, slice thickness: 8 mm, repetition/echo time: 4.6/1.3 ms, flip angle: 15–20°, matrix: 224 × 192.

All CMR studies were analysed off-line on the Advantage Workstation (GE Healthcare, Milwaukee, USA) with a dedicated software (MASS 6.1, Medis, Leiden, Netherlands) by an experienced CMR reader (A.B.) blinded to all other patient data. LV and RV volumes, mass and global function were calculated on SA cine images.

LV volumes were measured in each cardiac phase and plotted as a function of time to generate a V/t curve (Fig. 1). The pattern of the systolic phase of global V/t curves were determined by 2 independent expert CMR readers (G.D.A. and A.B.) blinded to all other CMR and clinical data. The NP consisted in a progressive, rapid reduction of LV volume with a well-defined, smooth peak, closely recapitulating the changes of volumes during systole. Conversely, the WP could display the following morphologies: “double peak” (two systolic peaks), “saw-tooth” (multiple peaks) or “flat” (large systolic plateau) (Fig. 1). A perfect inter-observer agreement (k = 1) was found in the identification of the WP or NP pattern. Even the intra-observer agreement during 2 repeated examinations 1-month apart was complete (k = 1). All healthy controls were evaluated in the same blinded fashion and found to have a NP.

The presence and extent of LGE were determined on short-axis images by detecting areas of myocardium with signal intensity ≥ 6 standard deviations above remote, non-enhanced myocardium [27, 28]. The maximal dV/dt ratio during the systolic phase and the contractility index (maximum rate of change of pressure-normalized stress: dσ*/dt_max_, where σ* = σ/P, and σ and P are circumferential stress and pressure, respectively) were calculated as indices of LV contractility [29].

For a regional analysis of LV systolic kinesis, the endocardial contours of all cardiac phases was divided into 6 equiangular segments at the basal and mid-ventricular levels and 4 equiangular segments at the distal level; the systolic dyssynchrony index was calculated as the standard deviation of the regional times to peak volume change [4]. V/t curves were derived in 16 LV regions (according to the American Heart Association/American College of Cardiology segmentation and excluding LV apex) [30], and the end-systolic peak was identified in each regional V/t curve. Maximal dyssynchrony time was defined as the temporal difference between the end-systolic peaks of the segments with the earliest and the latest peak; it was expressed both in seconds and as a percentage of the RR interval.

### Follow-up

All patients received optimal medical therapy, and CRT with defibrillation (CRT-D) when indicated [14, 15]. Results of CMR examination were not used to guide lead placement. In the absence of standardized criteria [31], the response to CRT was defined as improvement in at least 1 New York Heart Association (NYHA) class together with any increase in LVEF and ≥ 10% decrease in LV end-systolic volume at TTE after 3 months. The outcome status was assessed in December 2020 based on electronic health records (EHRs) or phone interviews with patients, relatives, or general practitioners; specifically, phone interviews were performed for all patients who did not have adverse outcomes recorded in the EHRs. The endpoint was a composite of cardiovascular death, HF hospitalization, or appropriate defibrillator shock; patients were censored at the time of the first event.

### Statistical analysis

Statistical analysis was performed using IBM SPSS Statistics (version 22, 2013). Normal distribution was assessed through the Kolmogorov–Smirnov test; variables with normal distribution were presented as mean ± standard deviation, while those with non-normal distribution as median and interquartile interval. Differences between groups were tested through the Mann–Whitney U test, and categorical variables were compared by the Chi-square test with Yates correction. In Kaplan–Meier analysis, survival was compared through the log-rank test (Mantel-Cox). Predictors of the composite endpoints were searched through univariate and bivariate Cox regression analysis; the “one-in-ten” rule was followed to avoid model overfitting [32]. Two-tailed p values < 0.05 were considered as significant.

## Results

### Population characteristics and correlates of the two V/t curve morphologies

Patients (n = 101) were aged 66 ± 11 years, 55% were males, and LVEF at CMR was 29% (25–35). The estimated glomerular filtration rate (eGFR) was 60 mL/min/1.73 m^2^ (46–80), and NT-proBNP levels were 1,060 ng/L (445–1994). Patients were on optimal medical therapy with beta-blockers, angiotensin-converting enzyme inhibitors or angiotensin-receptor blockers, mineralocorticoid receptor antagonists (MRA), in the absence of contraindications (Table 1). No patient had a device at baseline, and all patients were in sinus rhythm.

**Table 1:** Population characteristics

	All patients n = 101	Narrow pattern n = 29	Wide pattern n = 72	p
Age (years)	66 ± 11	63 ± 14	67 ± 9	0.314
Men, n (%)	55 (55)	15 (52)	40 (56)	0.726
BMI (kg/m^2^)	26 ± 4	26 ± 4	26 ± 4	0.991
LVEF CMR (%)	29 (25–35)	32 (28–39)	28 (25–33)	**0.015**
NYHA I/II-III-IV, n (%)	7/94 (7/93)	1/28 (3/97)	6/66 (8/92)	0.380
VEB grade IV-V Lown, n (%)	52 (51)	9 (33)	43 (59)	**0.003**
Diabetes, n (%)	24 (24)	1 (3)	23 (32)	**0.002**
Hypertension, n (%)	58 (57)	15 (52)	43 (60)	0.462
Hypercholesterolemia, n (%)	35 (35)	9 (31)	26 (36)	0.628
QRS width (ms)	150 (140–161)	150 (140–160)	150 (140–165)	0.468
Heart rate (b.p.m.)	68 (60–78)	65 (57–74)	70 (62–79)	**0.045**
Haemoglobin (g/dL)	13 (13–14)	13 (13–15)	13 (12–14)	0.226
eGFR (mL/min/1.73 m^2^)	60 (46–80)	72 (51–82)	60 (43–80)	0.252
NT-proBNP (ng/L)	1,060 (450–1,994)	493 (317–1,740)	1,338 (557–2,624)	**0.003**
NE (ng/L)	362 (270–585)	289 (202–412)	441 (304–625)	**0.003**
PRA (ng/mL/h)	1.4 (0.4–3.7)	1.2 (0.4–2.9)	1.4 (0.3–4.1)	0.731
Aldosterone (ng/L)	142 (76–224)	169 (80–220)	131 (74–225)	0.371
VO_2_/kg (mL/kg/min)	15 (12–17)	15 (12–18)	14 (12–17)	0.722
VE/VCO_2_	30 (28–36)	30 (28–35)	31 (28–36)	0.541
Beta-blocker, n (%)	988 (97)	27 (93)	71 (99)	0.140
ACEi/ARB, n (%)	95 (94)	29 (100)	66 (92)	0.109
MRA, n (%)	80 (79)	22 (76)	58 (81)	0.824

Significant p values are reported in bold

Variables with normal distribution were presented as mean ± standard deviation, while those with non-normal distribution as median and interquartile interval

*ACEi* angiotensin-converting enzyme inhibitor; *ARB* angiotensin receptor blocker; *BMI* body mass index; *LBBB* left bundle branch block; *MRA* mineralocorticoid receptor antagonist; *NE* norepinephrine; *NT*-*proBNP* N-terminal pro-B-type natriuretic peptide; *NYHA* New York Heart Association; *PRA* plasma renin activity; *PVC* premature ventricular complex; *VE*/*VCO*_*2*_ ventilation/carbon dioxide output; *VEB* ventricular ectopic beat; *VO*_*2*_ oxygen consumption

The systolic phase of the V/t curve displayed a NP in 29 patients, and a WP in 72. Mean QRS duration did not differ significantly between patients with a NP and those with a WP. However, patients with a WP had a higher heart rate, more frequent high-grade VEBs, and a more prominent neurohormonal activation, with markedly higher plasma NT-proBNP and norepinephrine (Table 1). End-systolic and end-diastolic diameters at transthoracic echocardiogram were greater in the WP group, while the grades of mitral regurgitation did not display significant differences (Table 2).

**Table 2:** Imaging findings in patients with narrow and wide pattern

	All patients n = 101	Narrow pattern n = 29	Wide pattern n = 72	p
TTE				
LVEF (%)	30 (25–35)	32 (28–39)	28 (25–33)	** < 0.001**
LVEDD (mm)	65 (59–70)	59 (54–66)	66 (60–70)	**0.001**
LVESD (mm)	54 (49–60)	50 (44–55)	55 (49–61)	** < 0.001**
E/e’	12 (9–16)	11 (9–16)	12 (9–17)	0.535
MR grades (mild, moderate, moderate-severe, severe)	48, 38, 7, 8	15, 10, 4, 0	33, 28, 3, 8	0.100
n (%)	(48, 38, 7, 8)	(52, 45, 13, 0)	(46, 39, 4, 11)	
TAPSE (mm)	20 (17–23)	19 (17–23)	20 (18–23)	0.737
CMR				
LVEF (%)	26 (21–34)	33 (27–40)	23 (19–29)	** < 0.001**
LVEDVi (mL/m^2^)	118 (92–145)	97 (58–131)	119 (98–150)	**0.013**
LVESVi (mL/m^2^)	86 (662–111)	72 (45–90)	93 (82–116)	**0.002**
LGE	52 (51)	12 (41)	40 (56)	0.192
LGE extent (% of LV mass)	2 (1.8–4.2)	2.0 (1.8–3.1)	2.1 (1.4–4.2)	0.080
Subepicardial LGE, n (%)	6 (12)	2 (17)	4(10)	0.790
Mid-wall LGE, n (%)	46 (88)	10 (83)	36(90)	0.500
Septal LGE, n (%)	15 (29)	3 (25)	12(30)	0.740

Significant p values are reported in bold

As all variables had a non-normal distribution, they were presented as median and interquartile interval

*CMR* cardiac magnetic resonance; *LGE* late gadolinium enhancement; *LVEDD* left ventricular end-diastolic diameter; *LVEDVi* left ventricular end-diastolic volume indexed; *LVEF* left ventricular ejection fraction; *LVESD* left ventricular end-systolic diameter; *LVESVi* left ventricular end-systolic volume indexed; *TAPSE* tricuspid annular plane systolic excursion; *TTE* transthoracic echocardiogram

### CMR findings

The numbers of patients with WP or NP are reported above; all healthy controls (mean age 64 years, 50% men) displayed a V/t curve morphology close to the NP. Patients with a WP had significantly greater LV indexed volumes and a lower LVEF, but no significant differences in terms of LGE prevalence, extent, or pattern (Table 2).

The WP pattern was associated with a longer maximal dyssynchrony time (absolute duration: 192 ± 80 vs. 143 ± 65 ms, p < 0.001; % of RR interval: 25 ± 11% vs. 8 ± 4%, p < 0.001). Even the systolic dyssynchrony index was higher in WP patients (13 ± 4 vs. 7 ± 3%, p < 0.001). The contractility index was lower in WP patients (2.6 ± 1.2 vs 3.2 ± 1.7, p = 0.045).

### Patterns of V/t curve for outcome prediction

Over a median 3.7-year follow-up (2.0–4.9), 7 patients died, 6 of them for cardiovascular causes. Eleven patients had defibrillator shocks because of life-threatening ventricular arrhythmias, and 15 patients were hospitalized because of worsening HF. Overall, the composite endpoint of cardiovascular death, HF hospitalization or appropriate defibrillator discharge occurred in 29 patients.

**Fig. 2:**
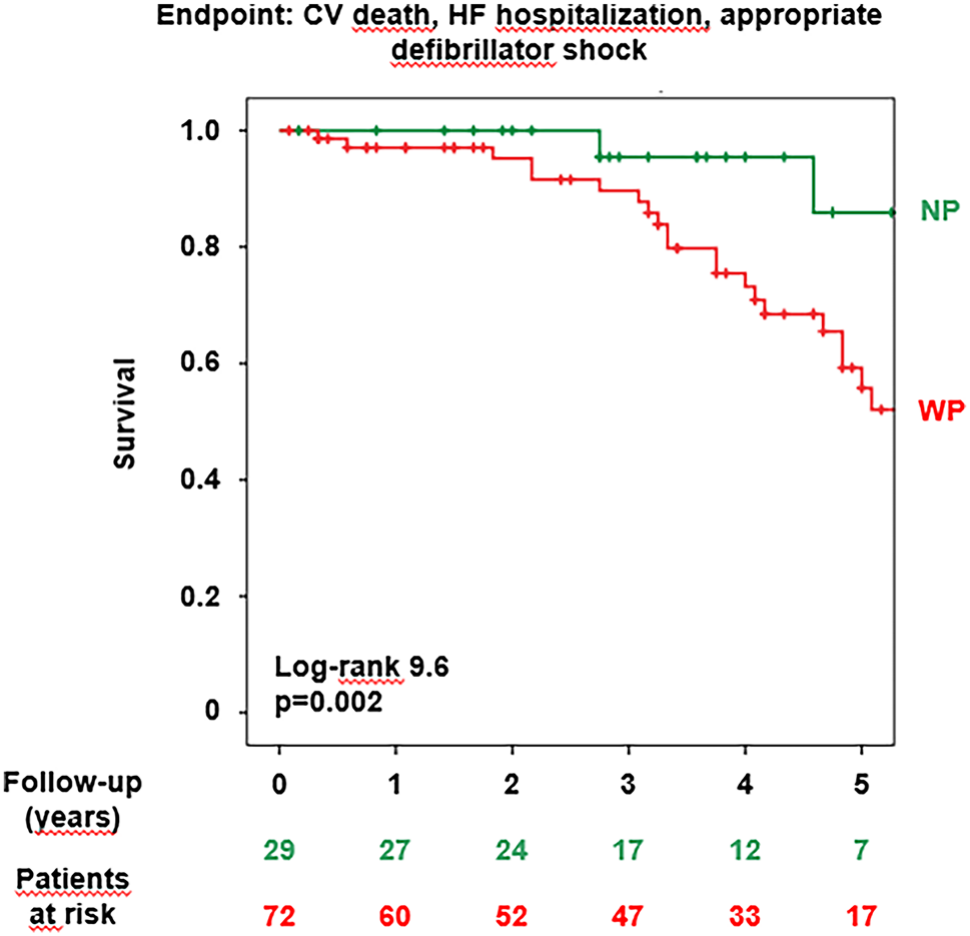
Patterns of the volume/time curve and event-free survival. *CV* cardiovascular; *HF* heart failure; *NP* narrow pattern; *WP* wide pattern

**Fig. 3:**
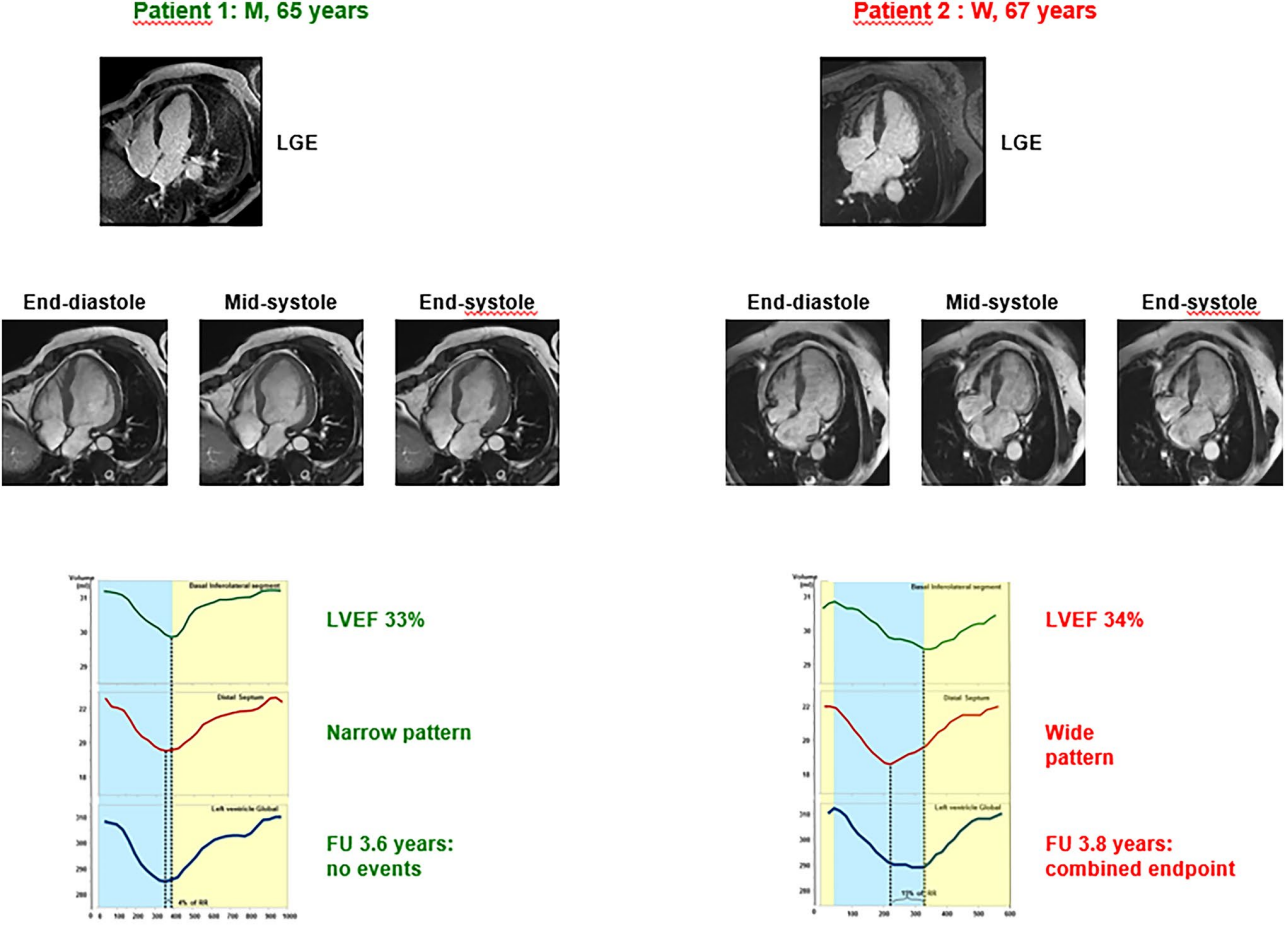
Patterns of the volume/time curve and risk of events. A 65-year-old man (*patient 1, left*) and a 67-year-old woman (*patient 2, right*) are presented. Late gadolinium enhancement (LGE) images showed small areas of mid-wall fibrosis in the interventricular septum in both cases, together with some subepicardial LGE in the lateral wall in patient 2. For each patient, 4-chamber acquisitions corresponding to end-diastole, mid-systole and end-systole are reported. Left ventricular ejection fraction (LVEF) and follow-up (FU) duration were similar, and LV end-diastolic volumes did not differ significantly (data not shown). Still, patient 1 had a narrow pattern and did not experience any event, while patient 2 had a wide pattern and had an event (heart failure hospitalization)

Patients with a WP had a significantly shorter survival free from the composite endpoint (Fig. 2). In other words, patients with a WP were more likely to experience an event during follow-up than those with a NP (Fig. 3). Among all characteristics listed in Tables 1 and 2, the following univariate predictors emerged: age, eGFR, NT-proBNP, LVESD at echo, LVEF at CMR, LGE presence, and WP (Table 3). The WP displayed an independent prognostic value from all the other univariate predictors, analysed by separate bivariate analysis because of the low number of events (n = 29; Table 4). Among the other variables, WP retains independent prognostic significance when evaluated against diabetes (HR 4.82, 95% CI 1.12–20.68, p = 0.034), which was significantly prevalent in the WP cohort (Table 1), and predicts worse outcomes in patients with HF [33].

**Table 3:** Univariate predictors of the composite outcome

	p	HR	95% CI
Age	**0.008**	1.06	1.02–1.11
Men	0.444	–	–
BMI	0.840	–	–
NYHA I/II-III	0.280	–	–
VEB grade IV-V Lown	0.991	–	–
Diabetes	0.602	–	–
Hypertension	0.294	–	–
Hypercholesterolemia	0.306	–	–
QRS width	0.263	–	–
Heart rate	0.064	–	–
Haemoglobin	0.278	–	–
eGFR	**0.021**	0.98	0.97–0.99
NT-proBNP	**0.047**	1.01	1.00–1.01
NE	0.547	–	–
PRA	0.614	–	–
Aldosterone	0.059	–	–
VO_2_/kg	0.406	–	–
VE/VCO_2_	0.779	–	–
Beta-blocker	0.252	–	–
ACEi/ARB	0.054	–	–
MRA	0.919	–	–
TTE			
LVEF	0.052	–	–
LVEDD	0.338	–	–
LVESD	**0.045**	1.01	1.01–1.02
E/e’	0.591	–	–
TAPSE	0.414	–	–
CMR			
LVEF	**0.048**	0.97	0.92–0.99
LVEDVi	0.712	–	–
LVESVi	0.608	–	–
LGE	**0.049**	2.16	1.01–4.66
LGE extent	0.055	–	–
Subepicardial/mid-wall/septal LGE	0.219	–	–
WP	**0.033**	4.81	1.14–20.37

Significant p values are reported in bold

Results from univariate Cox regression analysis are reported

*ACEi* angiotensin-converting enzyme inhibitor; *ARB* angiotensin receptor blocker; *BMI* body mass index; *CMR* cardiac magnetic resonance; *LBBB* left bundle branch block; *LGE* late gadolinium enhancement; *LVEDD* left ventricular end-diastolic diameter; *LVEDVi* left ventricular end-diastolic volume indexed; *LVEF* left ventricular ejection fraction; *LVESD* left ventricular end-systolic diameter; *LVESVi* left ventricular end-systolic volume indexed; *MRA* mineralocorticoid receptor antagonist; *NE* norepinephrine; *NT*-*proBNP* N-terminal pro-B-type natriuretic peptide; *NYHA* New York Heart Association; *PRA* plasma renin activity; *PVC* premature ventricular complex; *TAPSE* tricuspid annular plane systolic excursion; *TTE* transthoracic echocardiogram; *VE*/*VCO*_*2*_ ventilation/carbon dioxide output; *VEB* ventricular ectopic beat; *VO*_*2*_ oxygen consumption; *WP* wide pattern

**Table 4:** Independent prognostic value of the wide pattern (WP) for the prediction of cardiovascular death, heart failure hospitalization, appropriate defibrillator shock: bivariate Cox regression analysis

	Age	eGFR	NT-proBNP	LVESD	LVEF	LGE
WP	p = 0.015 HR 1.07 (95% CI 1.01–1.12)	–	–	–	–	–
	–	p = 0.025 HR 5.37 (95% CI 1.24–23.30)	–	–	–	–
	–	–	p = 0.012 HR 6.44 (95% CI 1.52–27.37)	–	–	–
	–	–	–	p = 0.024 HR 6.02 (95% CI 1.27–28.65)	–	–
	–	–	–	–	p = 0.044 HR 5.42 (95% CI 1.04–28.18)	–
	–	–	–	–	–	p = 0.011 HR 13.47 (95% CI 1.81–100.08)

p values, hazard ratio (HR) and the corresponding 95% confidence interval (CI) values for the WP as independent predictor of the composite endpoint are reported. The models for bivariate Cox regression analysis include alternatively age, estimated glomerular filtration rate (eGFR), N-terminal pro-B-type natriuretic peptide (NT-proBNP), left ventricular end-systolic diameter (LVESD), LV ejection fraction (LVEF), and late gadolinium enhancement (LGE)

*eGFR* estimated glomerular filtration rate; *HR* hazard ratio; *LGE* late gadolinium enhancement; *LVEF* left ventricular ejection fraction; *LVESD* left ventricular end-systolic diameter; *NT*-*proBNP* N-terminal pro-B-type natriuretic peptide

### Patterns of V/t curve and the prediction of response to CRT

After baseline CMR, 39 patients (28 with a WP, 11 with a NP) underwent CRT implantation (which in all cases was a CRT-D device). Despite their worse prognosis, patients with a WP responded more often to CRT than those with a NP (24/28 [86%] vs. 1/11 [9%] responders, respectively; p < 0.001). The prognostic benefit from CRT was evident also in the WP group, where patients on CRT had a better outcome (p = 0.020 at Kaplan–Meier analysis).

## Discussion

In patients with non-ischaemic systolic HF (LVEF < 50%) and LBBB, the systolic phase of the V/t curve showed 2 markedly different morphologies, namely a WP or NP. Patients with a WP displayed a worse HF status, more frequent high-grade VEBs, and a greater neurohormonal activation than those with a NP. The WP was also associated with more severe dyssynchrony and lower LV contractility. Patients with a WP had also a shorter survival free from cardiovascular events. Finally, among patients undergoing CRT implantation during follow-up, patients with a WP were more likely to respond to CRT.

An LBBB is found in about 20% of patients with HF [34], and is an expression of structural impairment of the myocardium. LBBB contributes to LV dysfunction by negatively affecting perfusion, systolic function and diastolic relaxation [35, 36]. Interestingly, studies exploring the clinical and prognostic correlates of LBBB have almost constantly performed a cumulative assessment of patients with this conduction disorder, despite the extreme variability in QRS duration and morphology, which suggests a similar heterogeneity in the determinants and consequences of LBBB [37, 38].

In this study we selected patients with non-ischemic cardiomyopathy to avoid the confounding factor of regional scarring altering mechanical contraction. We describe for the first time that the systolic phase of the V/t curve can display 2 morphologies, i.e. a NP or a WP. All patients had a LBBB, then a significantly impaired propagation of action potentials. Different patterns of impulse propagation may result in different morphologies of the V/t curve, without necessarily affecting total QRS duration. The NP demonstrates a relative preservation of the normal sequence of LV activation, while the WP is characterized by a markedly dyssynchronous LV contraction.

In our cohort, 29% of patients displayed a NP, and 71% a WP. The systolic dyssynchrony index was significantly higher in patients with WP. Accordingly, maximal dyssynchrony time was higher in patients with WP, on average 25% of the cardiac cycle, compared with 8% in patients with NP. In other words, ventricular activation was completed in a quarter of the entire cardiac cycle, compared with 8% of the cardiac cycle in patients with NP. Not surprisingly, LV systolic function was less effective in the WP subgroup, as demonstrated by a lower contractility index. In parallel, WP patients displayed greater diameters and volumes of the LV, demonstrating a more pronounced LV remodelling. An apparent discrepancy remains between the worse clinical outcome and the more frequent CRT response in WP patients compared to NP patients: it is possible that CRT might counteract disease progression in WP patients, but not enough to make their prognosis similar to NP patients. Further studies might address the differential prognostic impact of CRT in NP and WP patients.

CMR-assessed myocardial fibrosis has been repeatedly considered as a useful tool for risk stratification, and as a guide to treatment [39–41]. Different patterns of myocardial fibrosis could explain heterogeneity in LV conduction, manifesting as WP or NP. Nevertheless, no significant differences were found between WP and NP with regard to LGE presence; among patients with LGE, its extent or distribution pattern did not differ between WP and NP patients. Different LBBB morphologies thus seem to be related to a higher degree of LV remodelling rather than to different patterns of fibrosis, even though further larger studies are needed to address this point.

Calculation of another index of dyssynchrony, namely the CURE [8], requires complex post-processing which is not always available in CMR laboratories. Visual assessment of the systolic phase of the V/t curve may be a valuable and more viable alternative to these indices. Even the post-processing of tagging acquisitions is very complex and time consuming, whereas the V/t curves are automatically generated in the majority of current post-processing software for CMR without the need for regional evaluation. We found that WP was associated to higher systolic dyssynchrony index than the NP, confirming that the shape of V/t curve may be a simple way to detect mechanical dyssynchrony. Our method is simple, highly reproducible and commonly available because it requires only bSSFP short-axis images.

Several limitations must be acknowledged. First, this was a small, single-centre study, and confirmation on larger patient cohorts is warranted. Furthermore, although patients were managed according to Guideline recommendations, a limited number of patients received a CRT device [14]. This did not allow to perform a comprehensive assessment of predictors of response to CRT, including for example the presence and extent of myocardial fibrosis, or the correlates of such response, such as the changes in mitral regurgitation severity. Second, subgroup analyses considering specific aetiologies (including for example genetic dilated cardiomyopathy) were not performed. Third, temporal resolution is crucial to assess mechanical dyssynchrony, and CMR has a lower temporal resolution (ranging between 40 and 50 ms) than transthoracic echocardiography (< 10 ms). CMR assessment of dyssynchrony was not compared with an asynchrony study performed with an echocardiogram, which seems to predict response to CRT and outcome [42]. In addition to this comparison, further studies should examine the added value of a CMR assessment of dyssynchrony over an echocardiographic evaluation. Fourth, the small number of events required that a composite outcome measure be chosen, instead of single outcomes. Fifth, information from T1-mapping analysis was not available, although quantitative LGE evaluation allowed to search for the most evident and prognostically meaningful manifestation of an expansion of myocardial extracellular volume, i.e., myocardial scarring. Sixth, the possibility to draw conclusion with regard to the prediction of response to CRT is limited by the low number of patients.

In conclusion, in patients with non-ischaemic HF and LBBB, the presence of a WP in the systolic phase of the V/t curve identifies a subgroup of patients with greater LV dyssynchrony and a worse cardiac outcome, but a more frequent response to CRT. The systolic pattern of V/t curve might be used as a novel marker of mechanical dyssynchrony, and as a predictor of response to CRT.
